# m6A methylation profiling as a prognostic marker in nasopharyngeal carcinoma: insights from MeRIP-Seq and RNA-Seq

**DOI:** 10.3389/fimmu.2024.1492648

**Published:** 2024-12-12

**Authors:** Xiaochuan Chen, Wenqian Xu, Junping Pan, Hanxuan Yang, Yi Li, Xin Chen, Yingming Sun, Qinying Liu, Sufang Qiu

**Affiliations:** ^1^ Department of Radiation Oncology, Clinical Oncology School of Fujian Medical University, Fujian Cancer Hospital, Fuzhou, China; ^2^ Department of Radiation and Medical Oncology, Affiliated Sanming First Hospital of Fujian Medical University, Sanming, China; ^3^ Fujian Provincial Key Laboratory of Tumor Biotherapy, Clinical Oncology School of Fujian Medical University, Fujian Cancer Hospital, Fuzhou, China

**Keywords:** nasopharyngeal carcinoma, m6A modification, tumor immune microenvironment, prognosis, transcriptome sequencing

## Abstract

**Background:**

Nasopharyngeal carcinoma (NPC) is a type of malignant tumors commonly found in Southeast Asia and China, with insidious onset and clinical symptoms. N6-methyladenosine (m6A) modification significantly contributes to tumorigenesis and progression by altering RNA secondary structure and influencing RNA-protein binding at the transcriptome level. However, the mechanism and role of abnormal m6A modification in nasopharyngeal carcinoma remain unclear.

**Methods:**

Nasopharyngeal Carcinoma tissues from 3 patients and non-cancerous nasopharyngeal tissues from 3 individuals, all from Fujian Cancer Hospital, were sequenced for m6A methylation. These were combined with transcriptome sequencing data from 192 nasopharyngeal cancer tissues. Genes linked to prognosis were discovered using differential analysis and univariate Cox regression. Subsequently, a prognostic model associated with m6A was developed through the application of LASSO regression analysis. The model’s accuracy was verified using both internal transcriptome databases and external databases. An extensive evaluation of the tumor’s immune microenvironment and signaling pathways was performed, analyzing both transcriptomic and single-cell data.

**Results:**

The m6A methylation sequencing analysis revealed 194 genes with varying expression levels, many of which are predominantly associated with immune pathways. By integrating transcriptome sequencing data, 19 m6A-modified genes were found to be upregulated in tumor tissues, leading to the development of a three-gene (EME1, WNT4, SHISA2) risk prognosis model. The group with lower risk exhibited notable enrichment in pathways related to immunity, displaying traits like enhanced survival rates, stronger immune profiles, and increased responsiveness to immunotherapy when compared to the higher-risk group. Single-cell analysis revealed that malignant cells exhibited the highest risk score levels compared to immune cells, with a high-risk score indicating worse biological behavior. The three hub genes demonstrated significant correlation with m6A modification regulators, and MeRIP-RT-PCR confirmed the occurrence of m6A methylation in these genes within nasopharyngeal carcinoma cells.

**Conclusions:**

A prognostic model for nasopharyngeal carcinoma risk based on m6A modification genes was developed, and its prognostic value was confirmed through self-assessment data. The study highlighted the crucial impact of m6A modification on the immune landscape of nasopharyngeal cancer.

## 1 Introduction

Nasopharyngeal carcinoma is a malignant tumor originating from the mucosa of the nasopharynx, with a notably high occurrence in specific areas, especially in Southeast Asia and South China. The causes of nasopharyngeal cancer are not completely known, but they are linked to multiple elements such as genetics, environmental influences, and viral infections. Due to its insidious early symptoms, it is often detected at a middle to late stage, posing a great challenge to treatment (1). Thus, identifying the new marker is crucial for the early diagnosis and treatment of NPC. Over the past few years, advancements in genomics and transcriptomics have led scientists to increasingly recognize the significant impact of epigenetic changes on cancer progression. N6-methyladenosine (m6A) modification, a prevalent RNA alteration, significantly influences gene expression, RNA processing, and protein synthesis by modifying RNA structure and function (2).

m6A modifications are added to RNA by m6A methyltransferase (“Writer”) and removed by m6A demethylase (“Eraser”) removal, and recognition and decoding by m6A recognition proteins (“Readers”). This modification system forms a dynamic equilibrium that regulates multiple biological processes such as RNA stability, transcription, translation, splicing, etc (3). Growing amounts of evidence suggest that m6A modification plays a crucial role in controlling tumor development, resistance to chemotherapy, response to immunotherapy, and prognosis (4–7). It has been demonstrated that m6A modification is significantly linked to the onset, spread, and progression of tumors (8, 9). Additionally, m6A modification is essential in the complexity and diversity of the tumor microenvironment (TME) (10, 11). The interaction between m6A modification and the TME influences the biological activities of cancer cells, immune cells, and stromal cells, affecting tumor initiation, progression, and treatment responses (12–14). Grasping the relationship between m6A modification and the tumor microenvironment is crucial for creating effective treatments and predicting outcomes. While certain studies have highlighted the involvement of m6A modifications in cancer development, advancement, and therapy response, the majority of contemporary research is mainly centered on m6A regulatory proteins. The comprehensive study of how m6A-modified genes interact in nasopharyngeal carcinoma (NPC) and their effects on prognosis and the immune environment is still not well understood.

This research sought to combine m6A methylation histology with transcriptome data to pinpoint genes experiencing m6A methylation changes in nasopharyngeal carcinoma. The aim was to develop a predictive risk model utilizing m6A modification-associated genes to support treatment decisions for nasopharyngeal carcinoma patients and to investigate the model’s influence on the immune microenvironment.

## 2 Materials and methods

### 2.1 Patient samples

For transcriptome sequencing, tumor tissues from 192 nasopharyngeal cancer patients and normal tissues from 19 healthy individuals were collected from those diagnosed and treated at Fujian Provincial Cancer Hospital between January 9, 2015, and June 2, 2016 (in-house cohort). Additionally, tumor tissues from 3 nasopharyngeal cancer patients and non-tumor tissues from 3 healthy individuals were collected in 2023 for m6A methylation modification sequencing. Eligible participants included those newly diagnosed with nasopharyngeal carcinoma, undergoing standard radiotherapy, aged 18 or older, possessing normal blood, kidney, and liver functions, and free from other malignancies. Every patient gave their written consent after being informed. The Ethics Committees of both Fujian Cancer Hospital and Fujian Medical University Cancer Hospital granted approval for the research (approval code SQ2019-035-01). For future RNA extraction, tissue specimens were preserved in liquid nitrogen.

To confirm the reliability and relevance of the data in this study, NPC RNA-seq data from the GEO database (https://www.ncbi.nlm.nih.gov/geo/, GSE102349) were chosen as an external validation cohort. To assess the predictive effect of the risk model on immunotherapy efficacy, we downloaded the NSCLC-GSE126044 immunotherapy dataset from the GEO database. The model’s precision at the single-cell level was confirmed using the GSE150430 dataset, which also facilitated the investigation of cell-ligand receptor interactions within the NPC immune microenvironment.

### 2.2 m6A sequencing and processing of sequencing results

Hangzhou Lianchuan Biological Information Technology Co. handled the RNA extraction and the creation of sequencing libraries. The broken RNA was split into two sections. Initially, the sample was incubated for two hours at 4°C with an m6A-specific antibody.202003, Synaptic Systems, Germany) in immunoprecipitation buffer (50 mM Tris-HCl, 750 mM NaCl, and 0.5% isobaric acid). Tris-HCl, 750 mM sodium chloride, and 0.5% IGEPAL CA-630. The latter section functioned as a control to directly build a standard transcriptome sequencing library. The m6A-seq Library (IP) and RNA-seq Library (input) were individually processed for high-throughput sequencing on the Illumina NovaSeq™ 6000 platform in 150 PE mode. For superior read quality, the sequences underwent additional filtering with fastp (version fastp-0.19.4, available at https://github.com/OpenGene/fastp). To align the reads of all samples with the reference genome, we used the HISAT2 software package (https://daehwankimlab.github.io/hisat2/, version: hisat2-2.2.1). To analyze m6A and transcriptome samples, peak detection software along with the R package exomePeak 1.8 were employed, identifying peak positions on the genome, measuring peak lengths, and calculating differences between groups. ChIPseeker 1.0 was employed for further analysis.

### 2.3 Prognosis-related model construction and validation

Screening for differential m6A modifier genes between healthy population and nasopharyngeal carcinoma tissues by exomepeak2 analysis (15).The threshold criteria met these two conditions: a fold-change greater than 2 and a p-value less than 0.05.To delve deeper into the pathways enriched by DEGs, we utilized Gene Ontology (GO) and Kyoto Encyclopedia of Genes and Genomes (KEGG) pathway analyses. A false discovery rate of <0.05 was set as a critical value. Subsequently, these genes were compared with those showing variations between healthy individuals and nasopharyngeal carcinoma patients in the in-house cohort. This comparison was used to develop a prognostic model for m6A risk through univariate Cox analysis and LASSO Cox regression. The R package ‘glmnet’ was employed to pinpoint genes with the most valuable prognostic biomarkers. A predictive risk score was formulated by linearly integrating the equation: $Risk\;score=\sum_{i=1}^{N}\left(exp*coef\right)$ , where ‘exp’ represents the gene expression value and ‘coef’ denotes the gene’s coefficient in the LASSO analysis.

To assess the predictive accuracy of our risk prediction model, we categorized the sample into high-risk and low-risk groups based on the median risk score. Survival analysis was conducted using the R package ‘survival’. This approach facilitates a comprehensive understanding of the complex regulatory network associated with m6A modifications and provides valuable insights for identifying promising targets in the development of novel immunotherapeutic strategies. Survival curves were compared using the Kaplan-Meier technique. Later, the R package ‘timeROC’ was utilized to analyze the receiver operating characteristic (ROC) for individuals who survived 1, 3, and 5 years in both the self-assessment data cohort and the validation cohort GSE102349.

### 2.4 Multidimensional immunity- and carcinogenesis-related estimates

To assess immune cell infiltration in various ways, we used several immunoscoring methods, such as TIMER and ssGSEA algorithms (16, 17). The Immunophenotyping score was estimated by the IOBR-R package (18). From earlier studies, we retrieved a set of 10 suppressive immune checkpoints with immunotherapeutic efficacy (19). A set of genes for tertiary lymphoid structure (TLS) was also obtained (20, 21).

### 2.5 Single-cell RNA-seq analysis

Additionally, this research employed Seurat (version 4.0.4) for the purposes of quality assurance, data reduction, and grouping of single-cell RNA sequencing data (22). The data were quality controlled, downscaled and clustered using Seurat (v4.0.4). To maintain data integrity, genes identified in less than three cells and cells with under 250 detected genes were omitted, and the proportion of mitochondrial genes was restricted to below 35%. Data were normalized using the logNormalize method. TISCH (http://tisch.comp-genomics.org/) offers comprehensive single-cell level cell type annotations. Subsequently, the ‘FindAllMarkers’ function was employed to detect marker genes within each cluster, utilizing a threshold of absolute log2-fold change (FC) ≥ 0.3 and requiring a minimum cluster fraction of 0.25.

### 2.6 Calculation of risk scores and analysis of intercellular communication in single-cell samples

For each individual cell sample from GSE150430, risk scores were determined using the Single Sample Gene Set Enrichment Analysis (ssGSEA) technique, utilizing the ‘GSVA’ and ‘GSEABase’ libraries in R. Similarly, the risk scores for each tumor in the GEO validation group were computed with the same ‘GSVA’ and ‘GSEABase’ packages. Leveraging single-cell data as a benchmark, we utilized a novel deconvolution method (CIBERSORTx) on bulk transcriptome datasets to quantitatively determine the cell type proportions within tumors in both the self-assessment and GEO validation cohorts. CellChat version 1.1.3 software was employed to deduce communication between cells through ligand-receptor interactions. Cell groups containing fewer than 10 cells were excluded from the intercellular communication analysis. Pairwise tests of communication probability values were performed to assess statistical significance.

### 2.7 Statistical analyses

Data analysis was conducted with R (version 3.6.1) and SPSS (version 25.0) software. For continuous variables, the Wilcoxon rank-sum test was utilized, while the chi-square test was applied to categorical variables.In every analysis, pairs of two-by-two reveal significant statistical differences. Symbols *, **, ***, and **** denote significance levels of less than 0.05, 0.01, 0.001, and 0.0001, respectively.

## 3 Results

### 3.1 m6A modifier genes are differentiated in nasopharyngeal carcinomas

**Figure 1:**
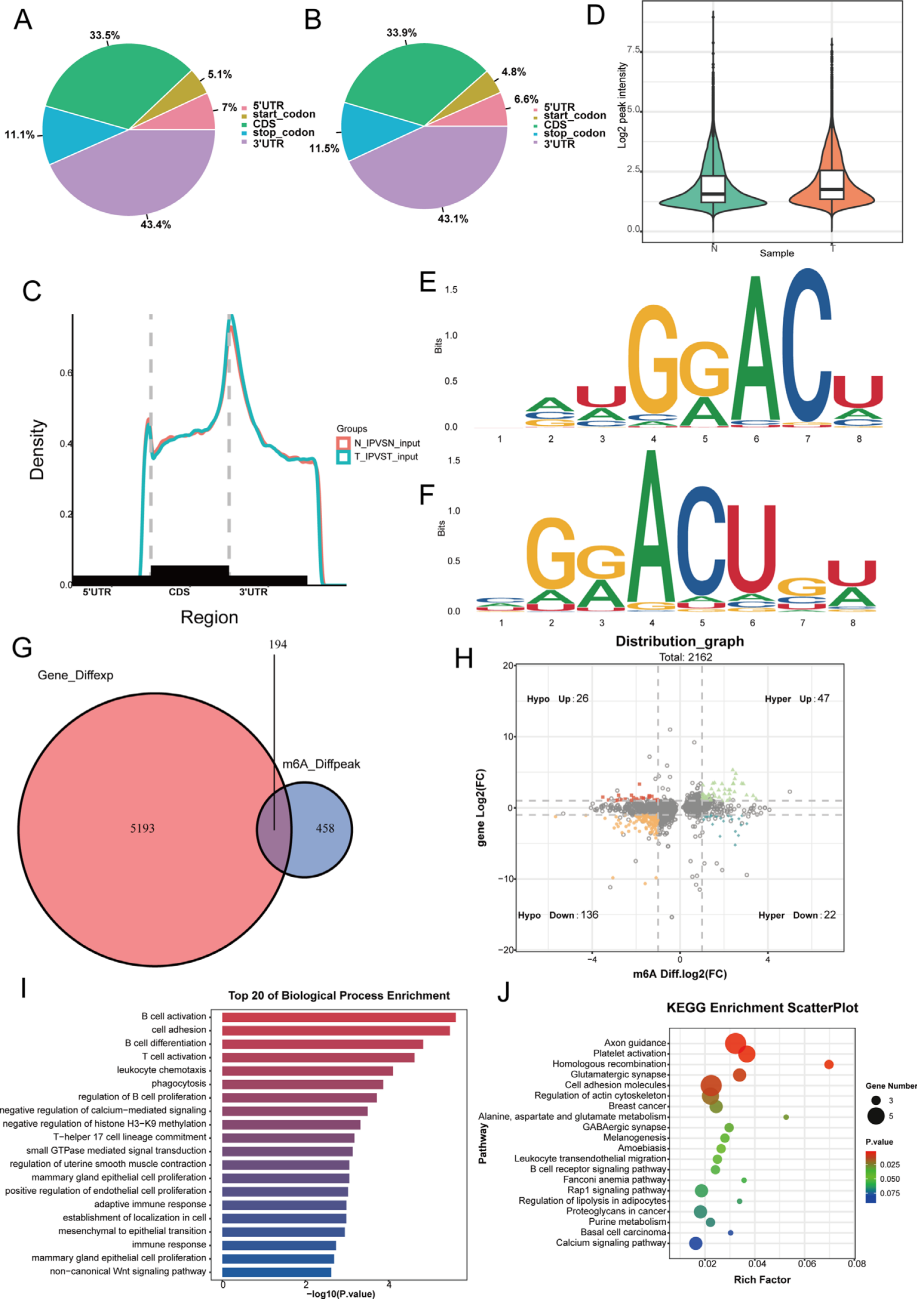
Analysis of m6a modifier profiles and identification of differentially expressed genes in nasopharyngeal carcinoma. **(A, B)** We use pie charts to count the distribution of peaks on gene functional elements between non-cancerous **(A)** and cancerous tissues **(B)**. **(C)** Density of differential m6A peaks along transcripts. Each transcript is divided into three sections: 5UTR, CDS, and 3UTR. **(D)** Levels of m6A methylation modification in tumor and non-tumor tissues. **(E, F)** Differential of the most conserved sequence motif in the m6A peak region. **(G)** Venn diagram showing differentially expressed genes undergoing m6a methylation modification between non-cancerous and cancerous tissues. **(H)** The four-quadrant diagram shows the changes in differentially methylated peaks. **(I, J)** The KEGG and GO enrichment pathway analysis of differential m6a methylated genes.

Analysis of m6A modification in three nasopharyngeal carcinoma samples and three normal nasopharyngeal tissue samples from Fujian Cancer Hospital revealed that m6A methylation predominantly took place in the coding sequences (CDS) and the 3h untranslated regions (3gionsl of both cancerous and non-cancerous tissues (**Figures 1A–C**). Compared with normal nasopharyngeal tissues, the levels of m6A methylation modification genes were higher in tumor patients (**Figure 1D**). Motif analysis revealed that RRACH methylation modification sites were present in both normal nasopharyngeal tissues and nasopharyngeal carcinoma tissues (**Figures 1E, F**). A total of 194 differential m6A methylation modification sites were identified in tumor and non-tumor tissues (**Figure 1G**), and the quadrant plot indicated that 65 differential m6A methylation modification genes were upregulated in nasopharyngeal carcinoma (**Figure 1H**). GO enrichment analysis indicated that the molecular roles of m6A modification genes were predominantly concentrated in signaling and immune response pathways, including B cell activation, T cell activation, and the inhibition of calcium-mediated signaling. Pathway analysis enriched by KEGG indicated that m6A modifier genes were predominantly involved in homologous recombination, cell adhesion molecules, and the B cell receptor signaling pathway (**Figures 1I, J**). The results indicate that m6A modification levels vary between cancerous and normal tissues and are intimately connected to the tumor immune microenvironment.

### 3.2 Risk modeling and validation

**Figure 2:**
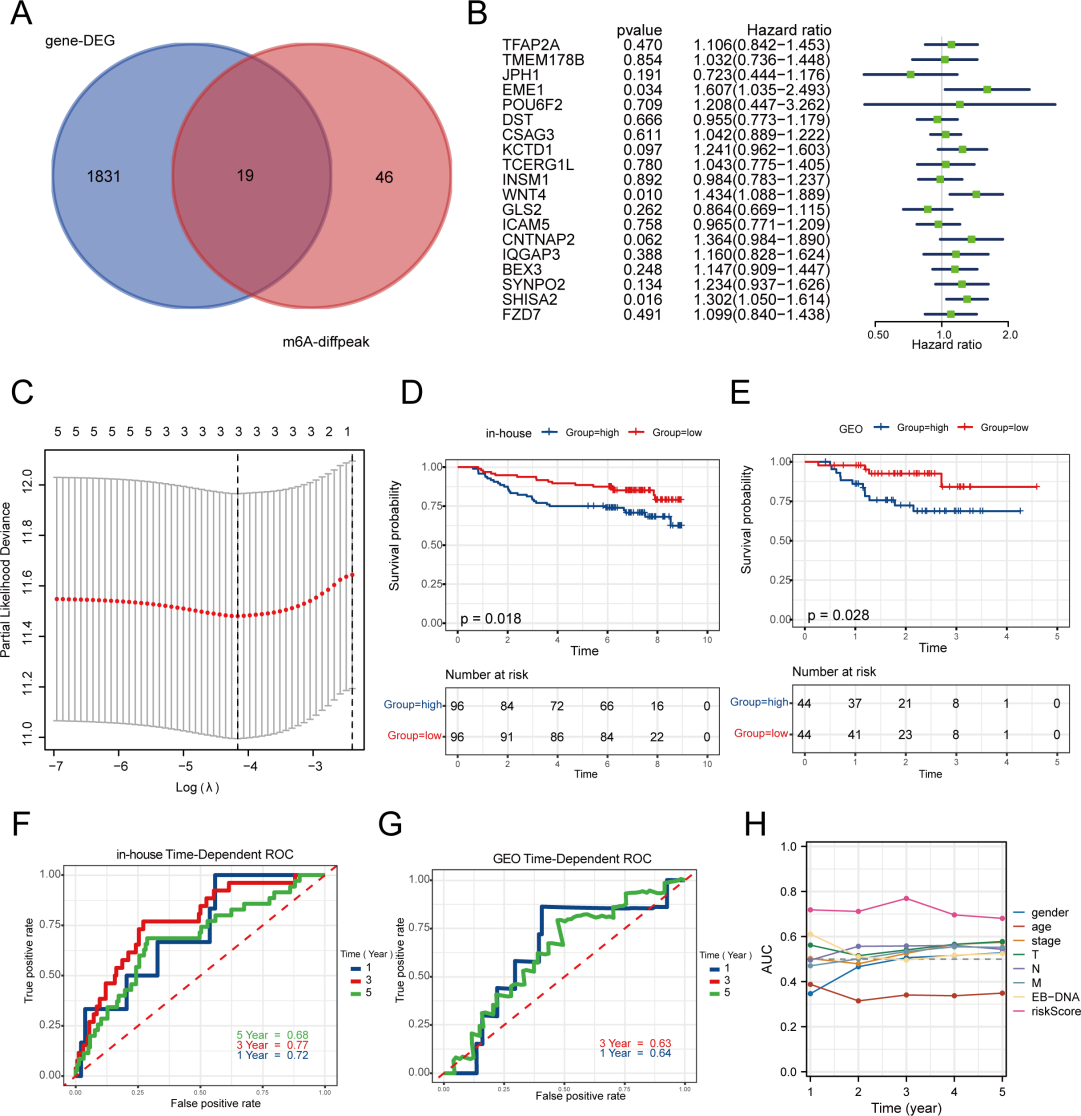
Construction and validation of a risk prognosis model for m6A related genes. **(A)** The intersection of m6A sequencing genes and 192 transcriptome data was used to screen for 19 19 m6A methylated genes upregulated in tumors. **(B)** Univariate Cox analysis was performed on these 19 genes with PFS. **(C)** Establishing prognostic biomarkers for three features (EME1, WNT4, SHISA2) identified in the in-house dataset using LASSO regression model. **(D, E)** In the in-house and GEO cohorts, low-risk group patients had a favorable PFS rate as opposed to those in the high-risk group formula. **(F, G)** The Receiver Operating Characteristic (ROC) curve for the 1-year and 3-year survival rates of in-house and GEO cohorts. **(H)** The ROC curve of clinical factors such as gender, age, stage, and risk score suggests that risk score has higher accuracy.

Differential genes in normal nasopharyngeal epithelial tissues and nasopharyngeal carcinoma tissues in the in-house cohort were further intersected with upregulated m6A methylation modifier genes in tumor tissues to identify 19 differential genes (**Figure 2A**); a one-way Cox analysis of progression-free survival (PFS) was performed using the survival R package to identify m6A modifier genes with prognostic significance (p-value < 0.05). This study identified 19 m6A modifier genes, including TFAP2A, TMEM178B, JPH1, EME1, POU6F2, DST, CSAG3, KCTD1, TCERG1L, INSM1, WNT4, GLS2, ICAM5, CNTNAP2, IQGAP3, BEX3, SYNPO2, SHISA2, and FZD7. Among these, three genes (EME1, WNT4, and SHISA2) had high prognostic significance (**Figure 2B**).

Based on these three central genes, the prognostic risk model (MRS) was established using the LASSO Cox regression model (**Figure 2C**). The dataset was split into high-risk and low-risk categories according to the median risk score. The in-house cohort confirmed that the high-risk category had a worse prognosis (**Figure 2D**). The high-risk group suggested a poorer prognosis, as was the case in the GEO validation cohort (**Figure 2E**). The MRS demonstrated strong predictive accuracy, achieving a 3-year ROC AUC of 0.77 (**Figure 2F**). Although the 3-year AUC of the validation cohort was only 0.63, it suggested the model’s stability (**Figure 2G**). Additionally, in comparison to gender, age, stage, and EBV-DNA, the model demonstrated a superior AUC (**Figure 2H**), suggesting that MRS serves as an independent prognostic indicator for predicting the survival of nasopharyngeal carcinoma patients and tailoring individualized treatment plans.

### 3.3 Enrichment pathways for risk model

**Figure 3:**
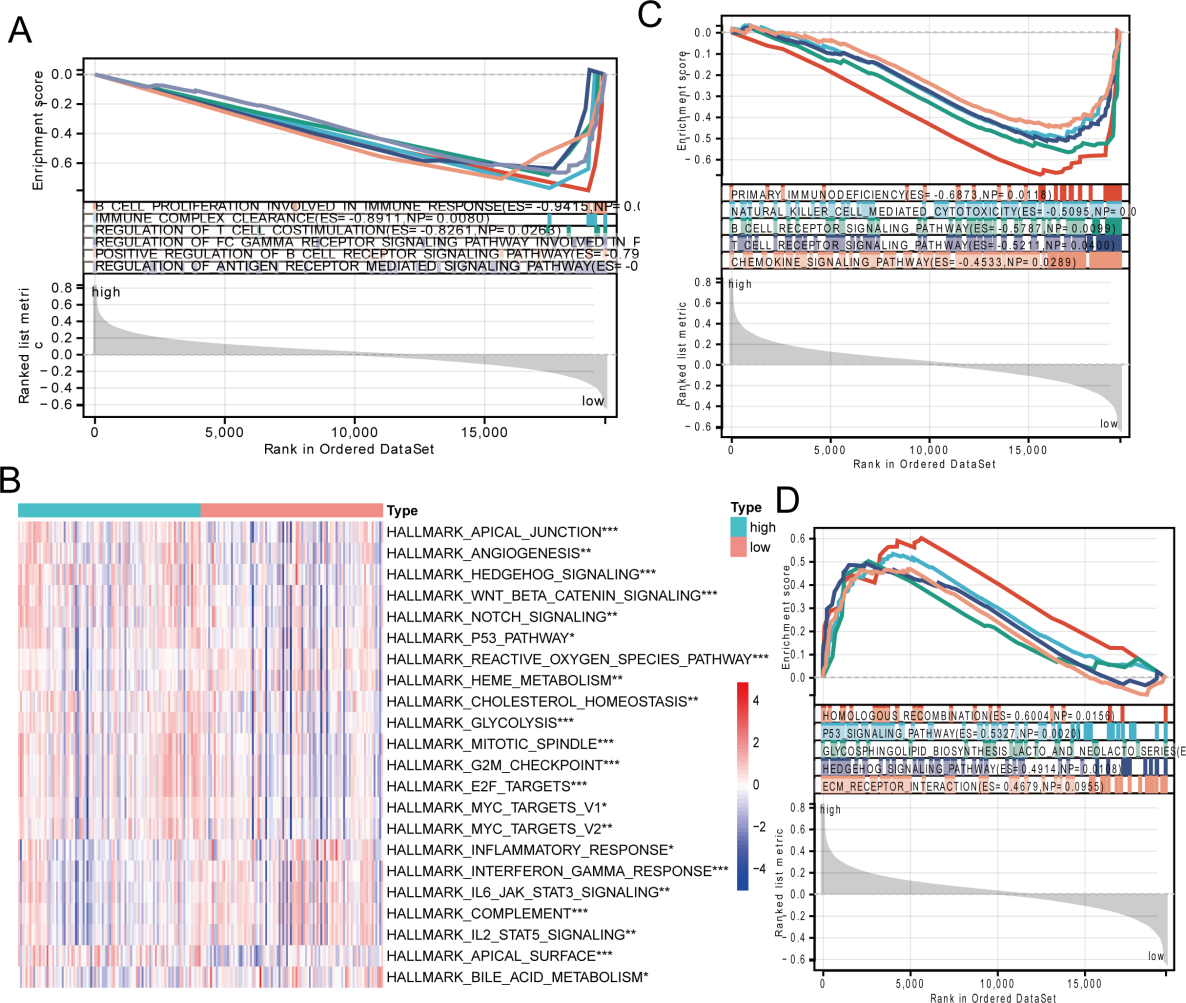
Signaling pathway enrichment analysis of risk models. **(A)** GO enrichment analysis of the low-risk group. **(B)** Heatmap showing HALLMARK pathway differences between high-risk and low-risk groups. **(C, D)** KEGG enrichment analysis in the low-risk group and high-risk group. * p < 0.05, ** p < 0.01, *** p < 0.001.

The pathways of gene enrichment suggested that the genes played roles in physiological processes. Gene Ontology (GO) enrichment analysis indicated that the genes in the low-risk category were predominantly associated with pathways related to cell growth, immune complex removal, and the modulation of T-cell co-stimulation, all of which play roles in B cell immune responses (**Figure 3A**). The heat map of the hallmark pathway and the KEGG enrichment analysis revealed that the high-risk group was predominantly enriched in pathways like homologous recombination, P53 signaling, glycolysis, and others. The low-risk category predominantly featured primary immunodeficiency, natural killer cell cytotoxicity, B-cell receptor signaling, and T-cell receptor signaling pathways (**Figures 3B–D**). To sum up, the immune microenvironment could be influenced by the low-risk group.

### 3.4 Assessment of the immune microenvironment

**Figure 4:**
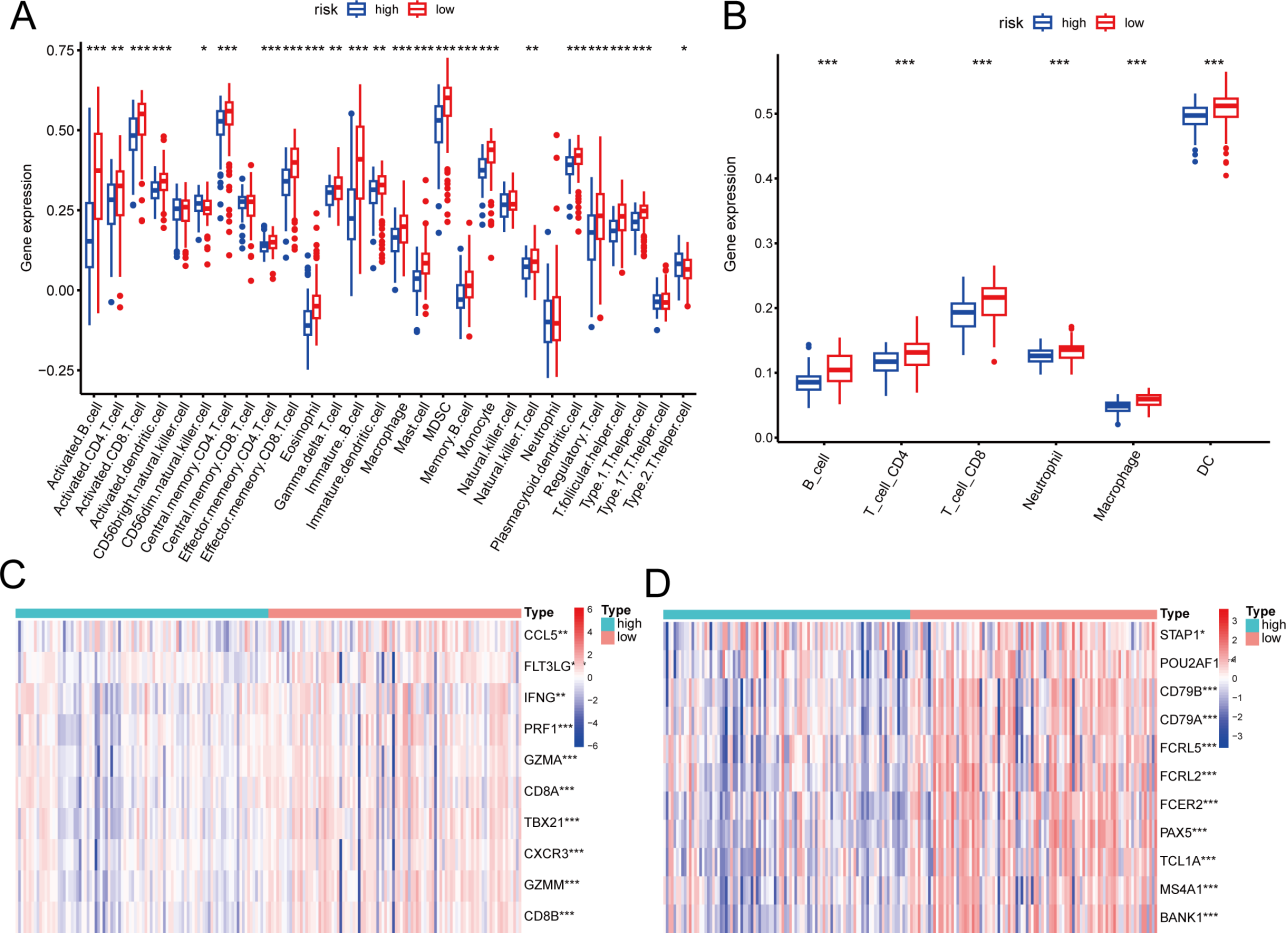
Association of the risk score with tumor immune microenvironment in nasopharyngeal carcinoma. **(A, B)** Differences in immune cell composition types between high-risk and low-risk groups by ssGSEA **(A)** and TIMER **(B)**. **(C, D)** Differences in marker genes between CD8+T **(C)** cells and B cells **(D)** in high-risk and low risk groups. * p < 0.05, ** p < 0.01, *** p < 0.001.

We assessed the variations in immune cell infiltration levels between groups at high and low risk. Using the ssGSEA technique, the makeup of the 28 immune cell types showed notable differences between the high- and low-risk groups. Nearly all immune cell infiltration levels were elevated in the low-risk group compared to the high-risk group, particularly for B cells and CD8+ T cells (**Figure 4A**). TIME analysis similarly validated these results (**Figure 4B**). Further analysis of marker genes for B cells and CD8+ T cells indicated a notable increase in their expression within the low-risk group (**Figures 4C, D**).

### 3.5 Predictive power of immunotherapy efficacy

**Figure 5:**
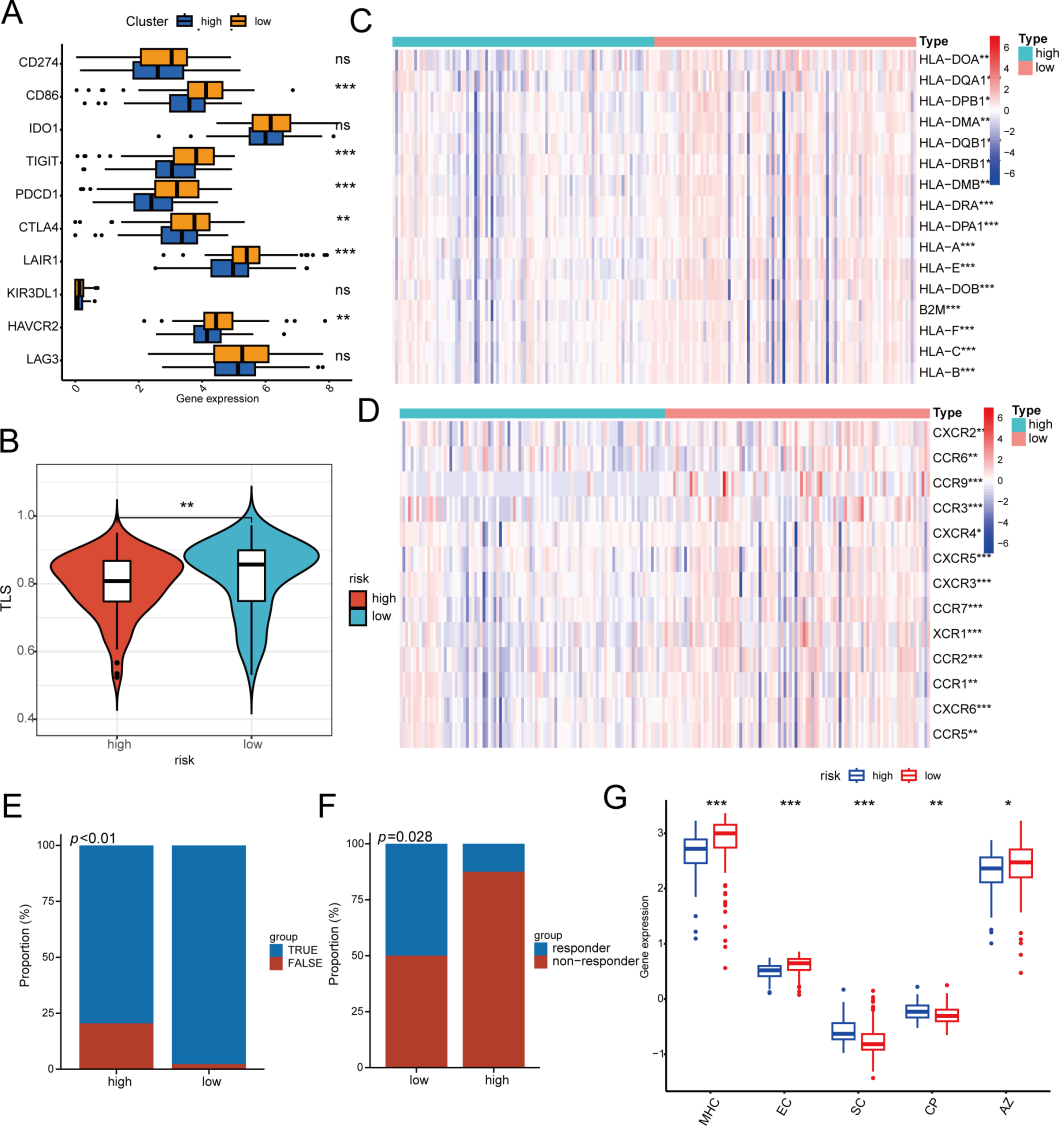
The response of immunotherapy of low- and high-risk groups. **(A)** The relationship between risk score and 10 inhibitory immune checkpoints. **(B)** Differences in TLS between high- and high-risk groups. **(C, D)** Differential Expression of Immune Cell Regulators and MHC in High and Low Risk Groups. **(E, F)** Patients in the low-risk group had higher immune responses in the cohorts of patients with nasopharyngeal carcinoma **(E)** and non-small cell lung cancer **(F)**. **(G)** Difference between low- and high-risk groups at ips score. MHC MHC molecules, EC effector cells, SC suppressor cells, CP immune checkpoints, * p < 0.05, ** p < 0.01, *** p < 0.001.

Moreover, a notable statistical disparity was observed in immune checkpoint inhibitors (CD86, PDCD1, TIGIT, CTLA-4, LAIR1, and HAVCR2) between the high-risk and low-risk categories (**Figure 5A**). Research indicates that B cells infiltrating tumors and tertiary lymphoid structures associated with tumors enhance the effectiveness of immunotherapy. We subsequently evaluated TLS scores and found that low-risk patients had higher TLS scores (**Figure 5B**), similar results were observed in many immune-related indices. In the low-risk patient group, the tumor enhanced immune cell activation and robust ligand-receptor interactions, providing the biological foundation for their favorable response to immunotherapy. There were notable differences in chemokine receptors and MHC molecules between the high- and low-risk groups. Specifically, receptors like CCR9, CCR3, and CXCR6 showed increased expression in the low-risk group, while the majority of MHC class II molecules exhibited decreased expression in the high-risk group, indicating a diminished capacity for antigen presentation and processing (**Figures 5C, D**). **Figure 5E** illustrates that, using the TIDE algorithm to evaluate nasopharyngeal cancer patients’ responsiveness to immunotherapy, the low-risk group experienced greater benefits from the treatment. Likewise, a uniform trend was seen in the group of patients undergoing immunotherapy for non-small cell lung cancer, with those in the low-risk category showing a stronger immune response (**Figure 5F**). The ips score also suggests this result (**Figure 5G**). To sum up, individuals classified as high-risk showed fewer advantages from immunotherapy and faced a poorer prognosis than those categorized as low-risk.

### 3.6 Single-cell analysis of immune environment and cell interactions

**Figure 6:**
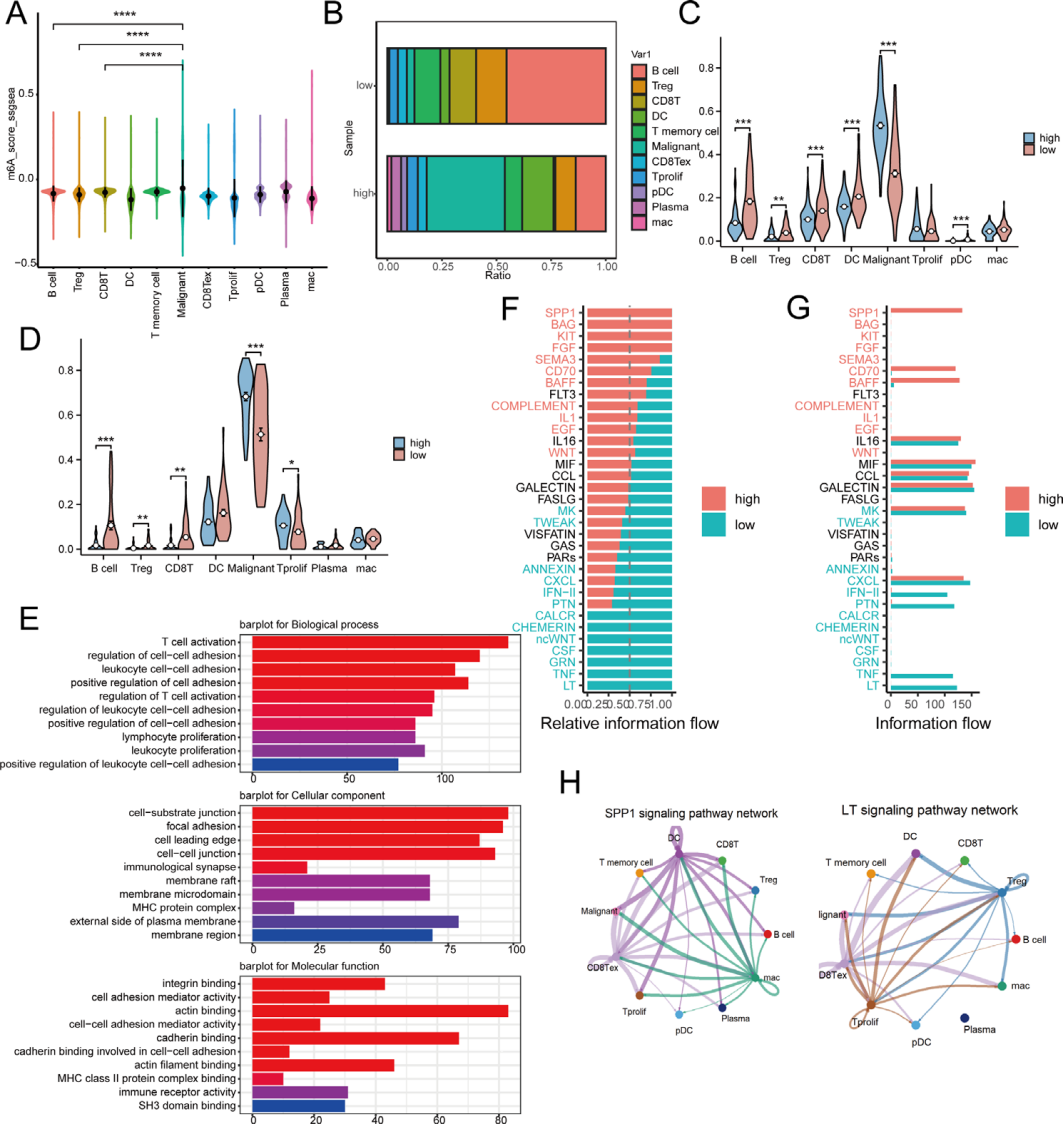
Risk model differences in immune landscapes and cellular communication at the single-cell level. **(A)** Risk scores for 11 different cell subgroup samples in the GSE150430 dataset. **(B)** The proportion of immune cell composition between high-risk and low-risk groups. **(C, D)** Detect immune cell infiltration in high-risk and low-risk groups in inhouse **(C)** and GEO **(D)** cohorts by CIBERSORTx tool. **(E)** The main pathways for accumulating differentially expressed genes between high-risk and low-risk populations. **(F, G)** Observing differences in active pathways between high-risk and low-risk groups. **(H)** SPP1 and LT signaling pathways in high-risk and low-risk groups. * p < 0.05, ** p < 0.01, *** p < 0.001, ****p < 0.0001.

In order to clarify the function of MRS within the immune microenvironment, we employed the single-sample gene set enrichment analysis (ssGSEA) technique to determine the risk score for each cell from GSE150430 (23). The findings indicated that in cancerous tissues, cells with greater malignancy exhibited elevated risk scores (**Figure 6A**). Based on median risk values, the samples were divided into high and low-risk categories. Low-risk samples exhibited a notably higher proportion of B cells and CD8 T cells compared to high-risk samples, which had a significantly greater percentage of malignant cells (**Figure 6B**). We then mapped the cell types of the single-cell dataset to in-house cohort and the GSE102349 cohort by the CIBERSORTX method. Predictably, cancerous cells showed elevated scores in the high-risk category in both the GEO database and transcriptome sequencing results, whereas CD8+ T cells and B cells were more abundant in the low-risk category (**Figures 6C, D**). These findings are consistent with previous studies indicating that higher risk scores predict poorer biological behaviors, and that low-risk scores correlate with a greater abundance of immune cells.

Subsequently, we conducted a functional analysis. The primary routes enriched with differential genes in both high-risk and low-risk categories were associated with cell adhesion and immune cell activation, indicating variations in response and immune resistance to distant metastasis between these groups (**Figure 6E**). Furthermore, the cellular signaling varied between the high-risk and low-risk groups. In the high-risk group, pathways such as CD70, SEMA3, FGF, KIT, BAG, and SPP1 were active, whereas in the low-risk group, pathways like LT, TNF, GRN, CSF, ncWNT, CHEMERIN, and CALCR were active (**Figures 6F, G**). **Figure 6H** illustrated the SPP1 in the high-risk category and the LT pathways in the low-risk category.

### 3.7 m6A methylation gene-related regulatory proteins

**Figure 7:**
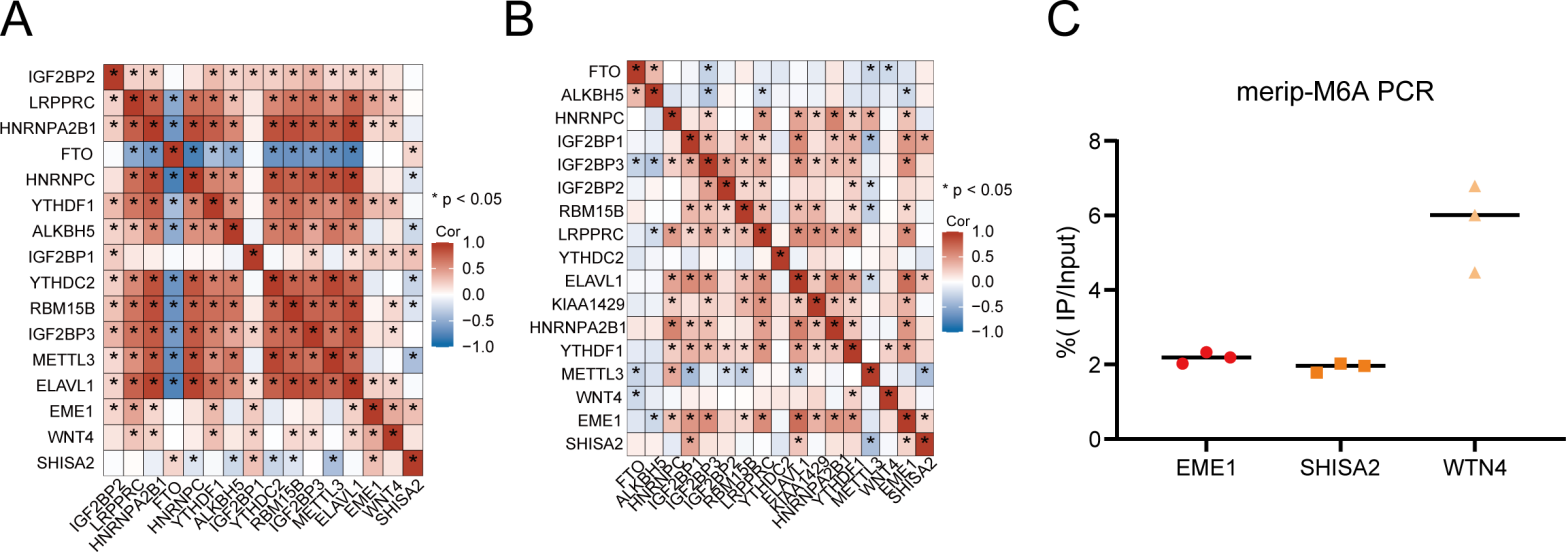
m6A modification levels of hub genes and their relationship with m6A regulatory proteins in nasopharyngeal carcinoma. **(A, B)** Three hub genes have strong correlation with m6A modification regulatory factors in the in-house **(A)** and GEO **(B)** cohorts. **(C)** MeRIP-PCR results of three hub genes in HK1 cell.

Correlation analysis of the three hub genes with m6A regulatory proteins in the GEO database and 192 cases of transcriptome sequencing revealed that EME1, WNT4, and SHISA2 were strongly correlated with most of the m6A modification regulators (**Figures 7A, B**). Subsequently, to verify whether the hub genes were methylated in nasopharyngeal carcinoma, we performed m6A methylation PCR on the three genes, and the results suggested that all three hub genes had high methylation levels in HK1 nasopharyngeal carcinoma cells (**Figure 7C**).

## 4 Discussion

This research underscores the crucial influence of m6A modifications on NPC tumor outcomes and the immune microenvironment, laying the groundwork for possible treatment approaches. Utilizing m6A and transcriptome sequencing, we identified three key prognostic genes (EME1, WNT4, SHISA2) with notable correlations, and developed an immune-related risk model for NPC. This model effectively forecasted progression-free survival in NPC and showed a strong connection with immune infiltration at both the transcriptome and single-cell levels.

In recent years, the exploration of methylation changes and the tumor immune microenvironment has become a prominent research area. RNA methylation is essential for maintaining internal balance and altering the metabolic landscape of the tumor microenvironment (TME), thereby influencing immune cell activity. One of the most prevalent RNA modifications is m6A methylation.m6A RNA methylation has been found to have multiple biological regulatory functions in cancer development and progression by regulating tumor immunity (7, 24, 25). Our research revealed that the m6A-based prognostic model for nasopharyngeal carcinoma risk showed a notable disparity in the immune microenvironment between high-risk and low-risk categories. The low-risk group exhibited a significant enrichment in various immune-regulatory pathways and demonstrated greater immune cell infiltration, particularly with B-cells and CD8+ T-cells, compared to the high-risk group. This indicates that individuals with low-risk ratings exhibit a heightened immune activity within the tumor’s surroundings, potentially leading to improved prognosis and therapeutic results.

Drugs that focus on PD-L1 and CTLA-4 are becoming more crucial in cancer therapy. The therapeutic impact of immune checkpoint inhibitors is directly influenced by the expression levels of PD-L1 or other immune checkpoints, thereby informing their clinical use. TLS is a lymphoid-like formation that typically develops in inflamed tissues. Recent research has indicated that tumor-infiltrating B cells and tumor-associated tertiary lymphoid structures are strongly linked to the effectiveness of immune checkpoint inhibitor treatments, offering new biomarkers for clinical decisions in immunotherapy. Our findings revealed that the low-risk group exhibited a higher count of memory B lymphocytes and elevated immune checkpoint expression, suggesting a higher likelihood of benefiting from immunotherapy. The precision of risk model forecasts was likewise confirmed across various immunotherapy groups. Beyond the topics covered here, further research is needed to explore the role of m6A methylation in various immune and immune-related cells, as well as its regulation in diverse biological processes and functions, such as metabolism, within immune cells, cancer cells, other stromal cells, and non-cellular components of the tumor microenvironment. This will help to fully understand the intricate regulatory network of m6A modifications and offer valuable insights for developing new immunotherapy approaches (26).

Three hub genes (EME1, WNT4, SHISA2) show strong correlation with m6A regulators and elevated levels of methylation modifications in nasopharyngeal carcinoma tissues. These genes play important roles in a variety of cancers, such as EME1 interacts with Mus81 to form a structure-specific nucleic acid endonuclease that maintains genome stability in mammalian cells (27) and is involved in regulating the development of cancers such as gastric cancer and breast cancer (28, 29), Wnt family member 4 (WNT4) is involved in regulating the progression of cancers such as gastric cancer and germline tumors (30, 31), SHISA2 is highly expressed in high-grade prostate (32). Nonetheless, the potential of these three genes with m6A modifications and their regulatory elements as biomarkers for diagnosing and predicting nasopharyngeal carcinoma, along with their specificity and sensitivity, still requires investigation (33).

Although we constructed a prognostic model for MRGs and provided novel insights to improve nasopharyngeal carcinoma management, this study has several limitations. Initially, additional research is required to confirm these results in broader and more varied patient groups, as well as to investigate the interplay between m6A modifications and other epigenetic elements. Understanding how m6A modifications interact with genetic, environmental, and viral factors in NPC could provide a more comprehensive picture of the disease and inform more effective prevention and treatment strategies. Moreover, additional immunological studies are required to investigate the possible mechanisms of the three key genes within the immune microenvironment of NPC.

## 5 Conclusions

In summary, this research underscores the crucial impact of m6A alterations on the prognosis of nasopharyngeal carcinoma and the immune environment. By establishing a risk-based prognostic model based on m6A modification genes, the study provides a valuable tool for predicting patient prognosis and tailoring therapeutic strategies. The distinct immune landscapes and pathway enrichments between high- and low-risk groups underscore the critical role of m6A modifications in NPC progression and treatment efficacy. These insights enhance our comprehension of NPC and open avenues for future studies and innovative therapies.

## Data Availability

The Partial datasets generated and analyzed during the current study are available in the GEO repository under accession number GSE102349 (https://www.ncbi.nlm.nih.gov/geo/query/acc.cgi?acc=GSE102349), GSE126044 (https://www.ncbi.nlm.nih.gov/geo/query/acc.cgi?acc=GSE126044) and GSE150430 (https://www.ncbi.nlm.nih.gov/geo/query/acc.cgi?acc=GSE150430). All original contributions discussed in this study are included in the article and its Supplementary Material. Additional information will be provided by the authors upon request, without any undue restrictions.
